# Policy congruence and advocacy strategies in the discourse networks of minimum unit pricing for alcohol and the soft drinks industry levy

**DOI:** 10.1111/add.15068

**Published:** 2020-05-30

**Authors:** Shona Hilton, Christina H. Buckton, Tim Henrichsen, Gillian Fergie, Philip Leifeld

**Affiliations:** 1 MRC/CSO Social and Public Health Sciences Unit, University of Glasgow, Glasgow, UK; 2 Institute of Law, Politics and Development, Sant’Anna School of Advanced Studies, Pisa, Italy; 3 Department of Government, University of Essex, Colchester, UK

**Keywords:** Alcohol, discourse networks, health, policy, public health, sugar-sweetened beverages

## Abstract

**Background and Aim:**

Public health policy development is subject to a range of stakeholders presenting their arguments to influence opinion on the best options for policy action. This paper compares stakeholders’ positions in the discourse networks of two pricing policy debates in the United Kingdom: minimum unit pricing for alcohol (MUP) and the soft drinks industry levy (SDIL).

**Design:**

Discourse analysis was combined with network visualization to create representations of stakeholders’ positions across the two policy debates as they were represented in 11 national UK newspapers.

**Setting:**

United Kingdom.

**Observations:**

For the MUP debate 1924 statements by 152 people from 87 organizations were coded from 348 articles. For the SDIL debate 3883 statements by 214 people from 175 organizations were coded from 511 articles.

**Measurements:**

Network analysis techniques were used to identify robust argumentative similarities and maximize the identification of network structures. Network measures of size, connectedness and cohesion were used to compare discourse networks.

**Findings:**

The networks for both pricing debates involve a similar range of stakeholder types and form clusters representing policy discourse coalitions. The SDIL network is larger than the MUP network, particularly the proponents’ cluster, with more than three times as many stakeholders. Both networks have tight clusters of manufacturers, think-tanks and commercial analysts in the opponents’ coalition. Public health stakeholders appear in both networks, but no health charity or advocacy group is common to both.

**Conclusion:**

A comparison of the discourse in the UK press during the policy development processes for minimum unit pricing for alcohol and the soft drinks industry levy suggests greater cross-sector collaboration among policy opponents than proponents.

## Introduction

The global rise in non-communicable diseases (NCDs) can be understood as ‘industrial epidemics’ driven at least in part by powerful corporations and their allies promoting products that are also disease agents [1]. Decades of mounting evidence on the tobacco industry highlighted its detrimental effect on health and brought about the introduction of upstream policies targeting price, marketing and availability. More recently, UK public health policymakers have turned their attention to upstream policy interventions targeting alcohol and sugar. There is growing evidence that the alcohol industry and ultra-processed food and drink industry use similar strategies to the tobacco industry to undermine effective public health policies [2–4].

Public health policy development is subject to a range of stakeholders presenting their arguments in the news media on the best options for policy action [5–7]. In this respect, the news media can be seen as important in contributing to agenda-setting [8] and in shaping public and policy opinion on the acceptability of public health policies [9–11]. Two recent examples of controversial pricing policy options that prompted intense media debates throughout the United Kingdom were minimum unit pricing (MUP) for alcohol and the soft drinks industry levy (SDIL). Both policy options were considered by the UK Government. However, while the SDIL was implemented throughout the United Kingdom in 2018, the introduction of MUP in England was placed on hold indefinitely in 2013, despite being included in the UK Government’s 2012 Alcohol Strategy [12]. Meanwhile, in June 2012, the Scottish Government passed the Alcohol (Minimum Pricing) Scotland Act 2012, paving the way for MUP in Scotland [13].

The MUP pricing policy targets the sale of cheap, high-strength alcohol to reduce alcohol consumption and related harms. After a failed legal challenge [14], in May 2018 a minimum price of 50p per unit was implemented in Scotland [15]. Arguments in support of MUP, appearing in the UK press, largely related to concerns about high levels of problem drinking; its effect on public health and public order; and a widespread belief that most of the alcohol that contributes to drunken behaviour is irresponsibly priced and sold [7,16]. Key opposing arguments in the debate positioned the policy as an illegal barrier to fair trade that would harm the economy and penalize responsible drinkers [7].

Public Health England’s report, ‘Sugar Reduction: The Evidence for Action’, highlighted the high levels of sugar consumption and associated health harms [17]. The report recommended a broad range of measures, including the introduction of a tax on high sugar products. In the March 2016 budget, the Chancellor of the Exchequer, George Osborne, announced the Conservative Government’s intention to introduce the SDIL [18]. They intended that the SDIL would encourage producers to re-formulate products with a reduced sugar content to avoid paying the levy [19]. Following a consultation period, the levy was introduced in April 2018 and set at 18p per litre on soft drinks with a total sugar content of 5 g or more per 100 millilitres, and 24p per litre for those with 8 g or more per 100 millilitres. The levy was to apply to all sugar-sweetened beverages except pure fruit juices (with no added sugar) and drinks with a high milk content. Key supportive arguments appearing in the UK press centred on the extent of the health harm caused by excess sugar consumption; that such a policy was a necessary government intervention as part of a package of measures; and that voluntary industry codes, such as the Public Health Responsibility Deal, had been ineffective [6]. Opposing arguments emphasized that industry was already taking voluntary action and playing an active role in health promotion, therefore further regulation was unnecessary;any form of taxation would be ineffective in tackling the complex problem of obesity; and such measures would cause economic harm to industry and the wider economy [6].

Successful implementation of ‘controversial’ health policies requires a high level of political commitment and support from advocacy stakeholders [20,21]. It has been argued that interest groups that present a united front may be more effective in having their preferred policy option adopted than if they work separately [22]. Indeed, Rasmussen and colleagues suggest that the likelihood of advocacy success increases when advocates publicly support each other’s position [23]. Hawkins & McCambridge suggest that a factor in the failure to implement MUP in England was that health advocates were initially underprepared and did not present consistent arguments for the policy in the media [21]. Conversely, the complex corporate relationships that exist between unhealthy commodity industries may represent an opportunity for strategic cross-industry collaboration and result in more coherent alignment of media messaging when seeking to influence policy development [24–26]. Smith and colleagues highlight the need for research to ‘better understand how processed food, soft drinks, and alcohol industries influence public, political, and policy debates’, in order to understand how to mitigate against industry messaging and successfully advocate for public health policy via the media [27].

This study seeks to address calls for research to compare stakeholder influencing activities across industry sectors [24,25,27]. We use discourse network analysis (DNA), a research method that allows the analysis and visualization of actor-based debates using network analysis, to explore the complex web of arguments, or discourse coalitions [28], that form when stakeholders seek to publicly influence government policy [29,30]. Previous studies have used DNA to describe the appearance of discourse coalitions in support of, and opposition to, MUP and SDIL [6,7]. In the recent commentary on Fergie *et al*., Schmidt highlights that this methodology is ‘likely to prove a particularly valuable tool for comparative research, allowing efficient, systematic, rigorous analysis to compare policy debates internationally and across multiple unhealthy products’ [31].

Here we aim to build on our previous DNA studies and use this methodology to compare stakeholders’ positions in the discourse networks across two pricing policy debates, MUP for alcohol and the SDIL, as represented in UK newspapers. The comparison of MUP and SDIL is an appropriate case study, as they are both examples of ‘sin taxes’ (pricing policies targeting products deemed harmful to society and individuals) [32,33]; intended to be UK-wide policies; and attracted a very public debate in the news media which, in turn, affected their chances of policy adoption. Specific research questions are: (i) what are the similarities and differences in the policy discourse networks’ composition and structure; (ii) how does the composition of coalitions differ between the two debates and what might this tell us about policy beliefs and advocacy strategies; and (iii) how do the arguments that polarize the coalitions differ?

## Methods

Pre-existing discourse network analyses on MUP [7] and SDIL [6] were employed as test cases to examine how DNA could be used as a comparative methodology. While the policy context was somewhat different for the two debates, both controversial policies drew significant media attention with clear polarization in stakeholder views, thus providing a useful case study. Additionally, although MUP was only finally implemented in Scotland, it was originally proposed as a UK-wide policy and included in the UK Government’s Alcohol Strategy [12].

We searched articles from 11 national UK newspapers, representing all political views and genres, in the months preceding and following key policy announcements: between May 2011 and November 2012 for the MUP debate; and between May 2015 and November 2016 for the SDIL debate. Stakeholder statements were identified and coded using the Discourse Network Analyzer (DNA) software [34], a qualitative content analysis software tool which combines category-based content analysis with network analysis [29,35]. Each coded statement consists of four variables: the person’s name, their organizational affiliation, the argument to which the subject refers (further called ‘concept’) and a binary qualifier indicating the stakeholder’s agreement or disagreement with the concept. Weighted one-mode networks of stakeholders were created for both debates and exported from DNA as stakeholder × stakeholder matrices, using the ‘subtract’ transformation with ‘average activity normalization’ [29]. These procedures create a network in which a tie connects any two stakeholder nodes if they agree (more than they disagree) with each other, regarding the concepts in the debate. The methods used to create the separate policy discourse networks are described in detail elsewhere [6,7]. To allow comparison between the pricing debates, common concepts were harmonized wherever possible. For example, ‘the policy will reduce consumption of the commodity’ was used in favour of ‘MUP will reduce consumption of alcohol’ and ‘the SDIL will reduce consumption of sugar-sweetened beverages’. Concepts that were unique to only one debate were not harmonized; for example, ‘industry plays an active role in public health promotion’ was specific only to the SDIL debate. For the MUP debate, 1924 statements by 152 people from 87 organizations were coded in 348 articles. For the SDIL debate, 3883 statements by 214 people from 175 organizations were coded in 511 articles. A total of 63 concepts were identified. Twenty-nine concepts were common to both debates, 17 unique to MUP, and a further 17 unique to SDIL. See Supporting information for a full list of concepts (Supporting information, Data S1) and stakeholder organizations (Supporting information, Data S2) appearing in each debate.

Networks were plotted in Visone (a software tool that allows the visualization and analysis of network structures in network data sets, such as those exported from the DNA software) [36]. Ties between actors represent common agreement or common disagreement with a specific concept or argument. A tie weight threshold equivalent to the 67th percentile was applied to the signed network to reduce ties to only relatively robust argumentative similarities and to maximize the identification of both network structures. The 67th percentile (equivalent tie weight thresholds 0.400 for MUP and 0.333 for SDIL) was selected to ensure that the networks could be directly compared. The Girvan–Newman edge-betweenness community detection algorithm (an algorithm to identify clusters, or discourse coalitions, in the network, i.e. groups of actors with a similar argumentative position) [37] was used to identify clusters of stakeholder subgroups with argumentative similarities within the discourse network. These clusters can be interpreted as discourse coalitions. The coalitions were then highlighted using blue hyperplanes, the different stakeholder types were visualized with common colours for both debates and the frequency of codes for stakeholders was represented by the size of the respective node. Network measures were used to compare the two networks and principal coalitions regarding: size—the total number of nodes (actors) in a network or cluster; density—a measure of connectedness of actors within a network cluster or the overall network, expressing the relative number of ties (i.e. the number of ties as a proportion of the theoretical maximum) [38]; and the E-I index—a measure of subgroup cohesion, i.e. how strongly aligned the actors are internally in any one cluster versus external alignment with other clusters [39]. The range for E-I index is –1 (all ties are internal to the coalition) to +1 (all ties are external to the coalition).

We examined the relative use of concepts in each debate by comparing the frequency with which they were used and the degree of agreement and disagreement. The concepts that were the most polarizing in each network were identified by: first, extracting the 15 most frequently used concepts for MUP and SDIL separately; secondly, calculating the ratio of agreement to disagreement for each concept; and finally, ordering them by this ratio. As such, the five most polarizing concepts were those with the highest ratio in each debate.

The primary research question and analysis plan were not pre-registered and thus the results should be considered exploratory.

## Results

### Overview

#### Research question (i) What are the similarities and differences in the policy discourse networks’ composition and structure?

[Correction added on 10 June 2020, after first online publication: The term ‘Respiratory quotient’ has been removed from the Results section in this version.]

**Figure 1:**
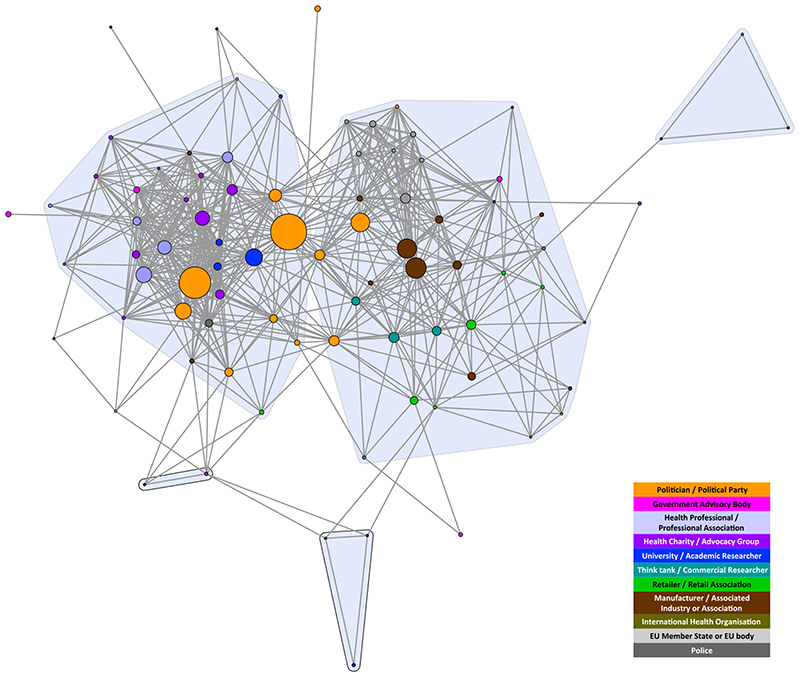
Minimum unit pricing (MUP) discourse network analysis (DNA) network showing stakeholder organizations colour-coded by stakeholder type. Tie weight cut-off at 67th percentile, i.e. < 0.400. Nodes sized by frequency in the debate. [Colour figure can be viewed atwileyonlinelibrary.com]

**Figure 2:**
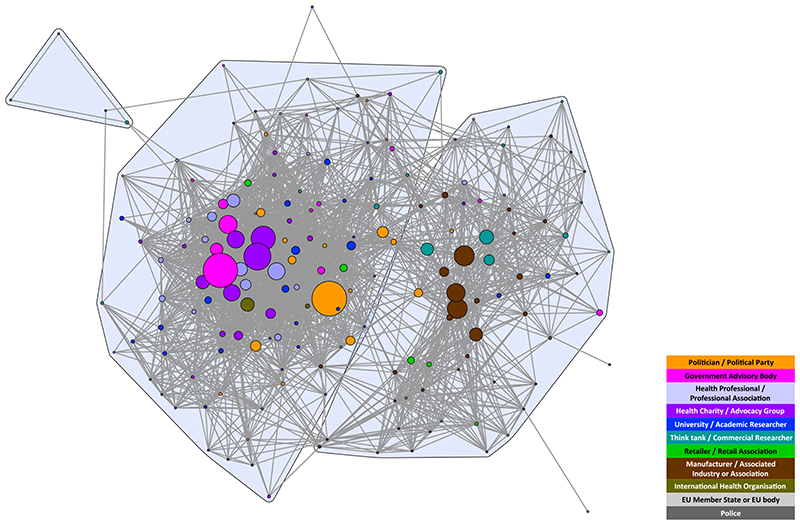
Soft drinks industry levy (SDIL) discourse network analysis (DNA) network showing stakeholder organizations colour-coded by stakeholder type. Tie weight cut-off at 67th percentile, i.e. < 0.333. Nodes sized by frequency in the debate. [Colour figure can be viewed atwileyonlinelibrary.com]

The composition of stakeholders in both networks was similar, reflecting the common interests of those participating in the debates. Both networks included politicians/political parties; government advisory bodies; health professionals/professional associations; health charities/advocacy groups; universities/academics; think-tanks/commercial researchers; retailers/retail associations; manufacturers/associated industries or associations; and international health organizations. The only stakeholder types that did not appear in both debates were European Union (EU) Member States/EU body and the police, which exclusively appeared in the MUP debate (Figs 1 and 2). Wine-producing EU Member States were particularly concerned about the legality of MUP, and the police highlighted MUP as a way of dealing with the violence resulting from ‘problem drinkers’, two issues that were not prominent in the SDIL debate. The detailed composition and characteristics of each network have been published elsewhere [6,7]. In this article, we focus on the comparison between the two networks and their respective coalitions.

The structure of both networks formed two discourse coalitions representing proponents and opponents of the policies. However, at the chosen tie-weight cut-off, the MUP coalitions are more distinct. Fewer stakeholders (total nodes) are engaged in the debate, with almost twice as many apparent in the SDIL network; 3.3 times as many in the proponents’ coalition and 1.7 times as many in the opponents’ coalition (Table 1). This reflects the greater number of vocal stakeholders in the SDIL debate, particularly in the proponents’ coalition. Additionally, the E-I index for proponents of SDIL is low compared with the other three coalitions (Table 1), indicating that members of this coalition were even more likely to agree with each other than with stakeholders outside the coalition, compared to the other coalitions.

### Composition of coalitions

#### RQ (ii) How does the composition of coalitions differ across the two debates, and what might this tell us about policy beliefs?

**Figure 3:**
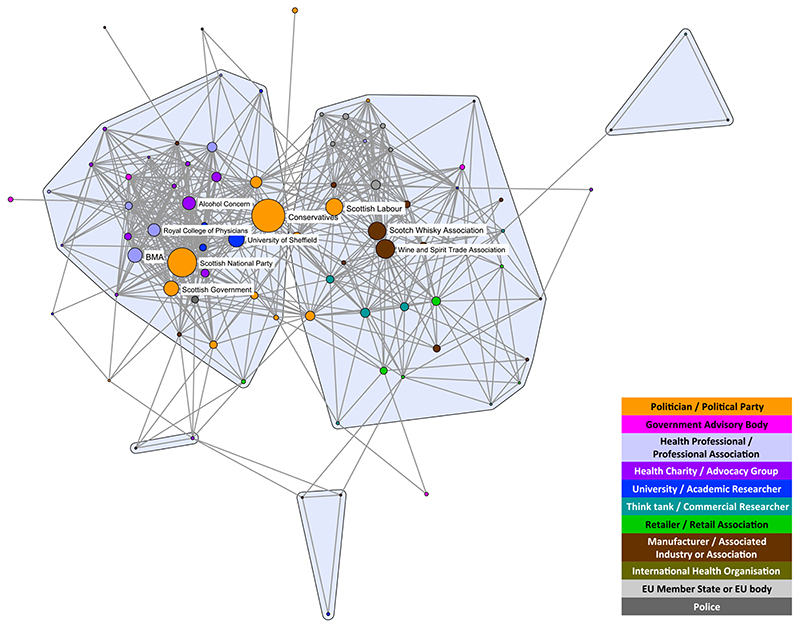
Discourse network analysis (DNA) network highlighting the 10 most active stakeholder organizations in the minimum unit pricing (MUP) debate. BMA = British Medical Association. [Colour figure can be viewed at wileyonlinelibrary.com]

**Figure 4:**
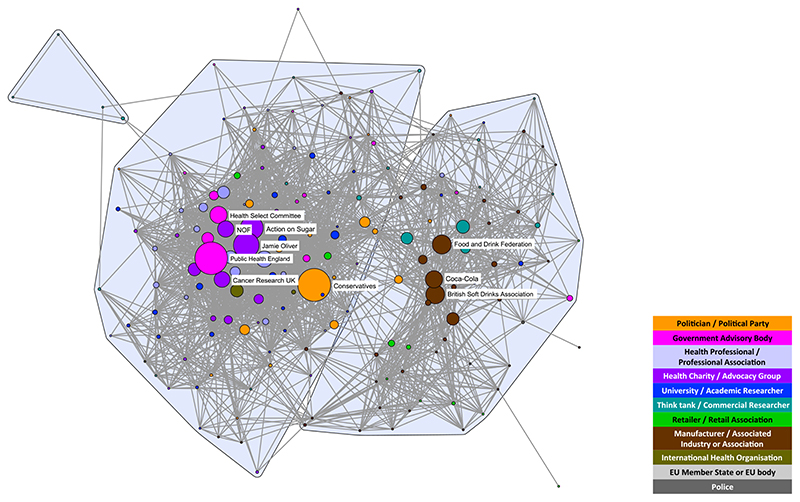
Discourse network analysis (DNA) network highlighting the 10 most active stakeholder organizations in the soft drinks industry levy (SDIL) debate. NOF = National Obesity Forum. [Colour figure can be viewed at wileyonlinelibrary.com]

Highlighting the 10 most active stakeholder organizations in each debate reveals that in both cases the commodity manufacturers and associated industry stakeholders (brown nodes) play prominent roles in opponents’ coalitions and are closely aligned with think-tanks and commercial researchers (teal nodes) (Figs 3 and 4). Associations representing manufacturers of the products under scrutiny are dominant spokespeople in both debates, in particular the Scottish Whisky Association and the Wine and Spirit Trade Association for MUP and the British Soft Drinks Association for SDIL. However, the SDIL network also features a prominent manufacturer (Coca-Cola) and an association representing related industries (the UK Food and Drink Federation).

The SDIL proponents’ coalition features active stakeholders from a wider range of public health advocates [government advisory bodies (pink nodes), particularly Public Health England, together with health charities and advocacy groups (purple nodes)] than seen in the MUP network. Six of the most active stakeholders are from these groups compared with only one (Alcohol Concern) for MUP. Other active stakeholders in the MUP proponents’ coalition are two professional associations (British Medical Association and the Royal College of Physicians) and one academic institution (University of Sheffield). While academic researchers are apparent in the SDIL network, they are not among the 10 most prominent stakeholders appearing in this debate.

Political stakeholders (gold nodes) appear among the two coalitions in both networks. However, only the Conservative party is among the most active stakeholders in the SDIL network, compared with four political parties in the MUP network [the Conservatives, Scottish National Party (SNP), Scottish Government and Scottish Labour]. This reflects the origins of MUP as an SNP policy targeting what was framed as a Scottish issue of harmful drinking. In both networks, the Conservative party is towards the middle of the networks. However, in both cases this does not reflect a brokering role, but either a change in ideology over the course of the debate (for SDIL, the Conservative shift in position in the middle of the period studied) or splits within the party on the issue (for MUP, prominent politicians openly taking opposing positions over the course of the period studied).

Despite similar patterns in the types of organizations making up the proponents’ and opponents’ coalitions across the two debates, only 30 organizational stakeholders are common to both (Table 2). This suggests that the debates are relatively sparsely connected to each other through common stakeholders, despite their topical similarity.

**Figure 5:**
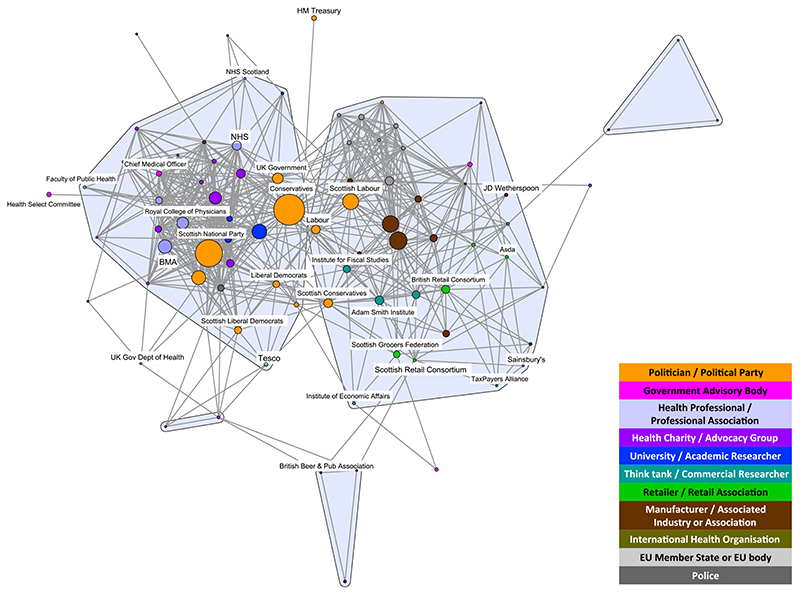
Discourse network analysis (DNA) network illustrating where stakeholder organizations common to both debates appear in the minimum unit pricing (MUP) network. BMA = British Medical Association. [Colour figure can be viewed at wileyonlinelibrary.com]

**Figure 6:**
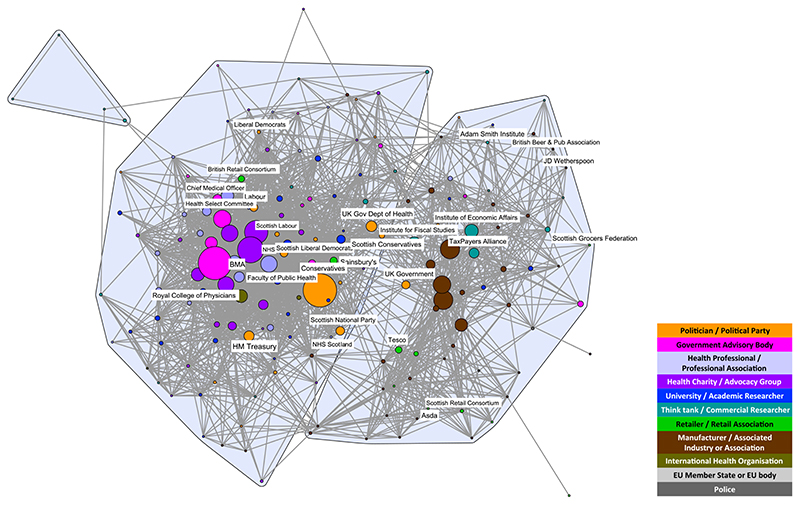
Discourse network analysis (DNA) network illustrating where stakeholder organizations common to both debates appear in the soft drinks industry levy (SDIL) network. BMA = British Medical Association. [Colour figure can be viewed at wileyonlinelibrary.com]

Apart from policymakers (political parties, government departments and advisory bodies), organizations from two other categories of stakeholders contribute to both the MUP and SDIL debates (Figs 5 and 6). Four think-tanks and commercial researchers (Adam Smith Institute, Institute of Economic Affairs, Institute for Fiscal Studies and the TaxPayer’s Alliance) and six retailers or retail associations (Asda, Sainsbury’s, Tesco, British Retail Consortium, Scottish Retail Consortium and Scottish Grocers Federation) appear in both debates. Think-tanks and commercial researchers (teal nodes) appear exclusively in the opponents’ coalitions, while the retailers and retail associations (green nodes) are spread across both coalitions in both debates. In relation to MUP, few retailers are central to the proponents’ coalition, unlike in the SDIL debate, where some retail stakeholders (e.g. Sainsbury’s and the British Retail Consortium) are integrated into the proponents’ coalition with strong belief ties to key policy proponents.

It is noteworthy that, in contrast, there were no health charities or advocacy groups common to both debates, despite a range of these organizations being very active and central to the proponents’ coalitions within each debate. Similarly, while universities and academic researchers appear in one or other debate, only the University of Birmingham is common to both.

### Polarizing arguments

#### RQ (iii) How do the arguments that polarize the coalitions differ?

Of the top five concepts that lead to the formation of coalitions in the two networks, two concepts are common to both (Table 3). ‘Policy is supported by the evidence’ is the most polarizing concept for both networks and ‘policy will reduce consumption of the commodity’ is the third and fourth most polarizing concept for MUP and SDIL, respectively. Three of the most polarizing concepts are unique to one or other of the debates: ‘policy will penalize responsible consumers’ for MUP; ‘industry is taking voluntary actions’ and ‘industry plays an active role in public health promotion’ for SDIL. Of note is the fact that the two most frequently cited arguments in the SDIL debate do not appear as significant polarizing concepts, i.e. ‘policy needed to address commodity problem’ and ‘commodity consumption causes health harm’. These concepts relate to the framing of the problem in relation to population-level health harm and the need for a policy response. Conversely, the two most frequently cited arguments in the MUP debate result in network polarization, i.e. ‘policy will reduce consumption of the commodity’ and ‘policy is illegal’. In contrast, these concepts relate to the framing of the solution and its probable effectiveness and legality. Thus, the most frequently cited arguments in the SDIL debate do not result in polarization of the network, suggesting a high degree of agreement about the extent of the problem resulting in more closely integrated coalitions.

## Discussion

There are calls for more nuanced analyses of stakeholder engagement in health policy development [24,27,31]. It has been suggested that research should compare stakeholders across multiple unhealthy products and related policies [31]. Using DNA methods, this study presents the first direct comparison of the discourse coalitions that were evident in the UK press during the policy development processes for MUP and the SDIL. Both networks show similarities in terms of structure, proponents’ and opponents’ coalitions and similar stakeholder types. However, important differences are revealed in terms of network size and complexity; the relative prominence, and lack thereof, of key stakeholders; subtle differences in the position of industry subsegments between networks; and the relative polarizing impact of frequently cited arguments.

Proponents of the pricing policies in both debates included public health, health charities, advocacy groups and academics. While these stakeholders were present in both debates, few specific organizations were common to both, suggesting that such proponents tend to make media statements focusing on their area of policy interest. While it is clear that policy advocates are already working across sectors; for example, in the guise of the Cross Party Group on Improving Scotland’s Health: 2021 and Beyond [40], and health alliances across the United Kingdom and internationally, this study suggests that they may not optimize their media messaging with regard to pricing policies. The World Health Organization (WHO) identifies such upstream policies as ‘best buys’ to tackle non-communicable diseases across all life-style factors [41]. There may be potential space for further cross-sector public health advocacy in support of pricing policies, by elevating the debate and presenting arguments across policy debates in support of their counterparts. Advocates could thus increase their chances of achieving policy congruence, as suggested by Rasmussen and others [23,42].

In contrast, opponents of regulatory pricing policies were present in both policy debates, specifically those with a vested interest in the economic impact of both policies such as retailers, representatives of licensed premises and commercial researchers. This structural similarity suggests industry stakeholders hold comparable discourse positions, supporting the idea of a common industry ‘playbook’, facilitated by public spokespeople, as suggested by Petticrew et *al*. [43]. The same four free market think-tanks and commercial researchers appear embedded in both opponents’ coalitions, closely tied to industry stakeholders, suggesting similar market justice rhetoric based on commercial ideology [44,45].

Comparing alcohol and tobacco strategies, Savell and colleagues suggest that there are commonalities, including both sectors providing skewed interpretations of evidence while also promoting voluntary codes, based on establishing themselves as acting responsibly in relation to health [4]. Our findings support this by suggesting that both sides focus on the availability and quality of evidence and this is the most significant polarizing argument in both networks. There may be an opportunity for policy advocates and academics to focus their advocacy efforts in the media on stressing the importance of weight of evidence, strength of evidence, source of evidence and how it is best used. Polarizing concepts appearing in the SDIL debate but absent in the MUP debate are ‘industry is taking voluntary action’ and ‘industry plays an active role in public health promotion’. This lends support to Nixon et al.’s findings that the food and drinks industry seeks to establish themselves as an exceptional case that should not be subject to the same controls as producers of other health-harming products, and is a key part of their corporate social responsibility rhetoric [6,46]. However, Collin et *al*. highlight the linkages that exist across tobacco, alcohol and ultra-processed food companies, positing the idea of a single unhealthy commodity industry requiring a consistent regulatory approach [2].

A key difference between the two networks is the number and distribution of associated industry stakeholders such as retailers and restaurants, with a greater number in the SDIL network, including the active voice of the UK Food and Drink Federation. Six key retailers are common to both debates but appear in different positions. For example, the British Retail Consortium and Sainsbury’s appear as proponents of SDIL and opponents of MUP, whereas Tesco occupies inverse positions. This, together with wider industry engagement in the SDIL debate, reinforces the need to clearly define industry subsegments and their policy positions, as suggested by Collin *et al*. [24]. Policy advocates may benefit from understanding the policy responses of multiple industry subsegments to effectively counter policy objections and leverage potential policy support.

One of the limitations of this study, which examines the debates as static networks, is that it does not allow analysis of subtle shifts over time. While the change in position of the Conservative Party in the SDIL debate was the only fundamental change in ideological position, there was an ongoing interplay of subtle shifts in emphasis and relative prominence of arguments over time in both debates. Future studies would benefit from comparing network development over time. Secondly, harmonizing the concepts for the two debates may have resulted in the loss of some nuanced arguments. However, the coders of the two debates worked together to ensure consistency and minimize this risk. Thirdly, the periods studied for each debate were 4 years apart: 2011–12 for MUP and 2015–16 for SDIL. The passage of time could have influenced stakeholders’ strategies and the nature of their responses to proposed fiscal policy. However, we chose these time-periods deliberately to examine the debates at similar stages of policy development. Finally, while we recognize the importance of the digital world of echo chambers, tailored information and micro-targeting, which means that social media plays an increasing role in influencing the policy agenda [47], traditional newspapers remain an important barometer of the current political agenda.

## Conclusion

In conclusion, this visualization of the discourse networks apparent in the debates on pricing policies spanning two unhealthy commodity industries may represent a manifestation of the underlying discursive strategies (manipulation or framing of a set of arguments by actors in order to achieve a certain goal) employed by policy stakeholders to influence policy makers and the public, via the news media. The network comparison is suggestive of greater cross-sector collaboration among policy opponents than proponents. Our analysis also suggests that, in seeking policy congruence, there may be a space for further cross-sector public health advocacy, by presenting arguments across policy debates in support of their counterparts. However, we recognize there are potential barriers to this model, not least resource constraints and the risk of mission creep for some public health advocates. Given the limited presence of academic institutions across the networks, and the importance of statements relating to evidence in polarizing both networks, we suggest that academics contribute more frequently on issues relating to evidence in policy debates. Finally, we suggest that DNA could usefully be applied to compare other policy debates over time and across countries, in attempting to tackle NCDs.

## Supplementary Material

Additional supporting information may be found online in the Supporting Information section at the end of the article.

**Data S1** List of concepts in each debate.

**Data S2** List of stakeholder organisations in each debate.

Data S1

Data S2

## Figures and Tables

**Table 1:** Network comparisons.

*Network measures*	*MUP*	*SDIL*
Whole network		
Total nodes	87	175
Total ties	617	2,463
Density	8%	8%
Proponents coalition		
Nodes	33	109
Total ties	365	1,900
Internal ties	287	1,739
External ties	78	161
Ties to opponents’ coalition	60	155
Density	27%	15%
E-I index	−0.57	−0.83
Opponents coalition		
Nodes	35	60
Total ties	301	715
Internal ties	231	558
External ties	70	157
Ties to proponents’ coalition	60	155
Density	19%	16%
E-I index	−0.53	−0.56

MUP = minimum unit pricing; SDIL = soft drinks industry levy

**Table 2:** Common stakeholders appearing in both networks.

*Stakeholder category*	*Specific stakeholders appearing in both networks*
Politician/political party	Conservatives/Labour/Liberal Democrats Scottish Conservatives/ Scottish Labour/Scottish Liberal Democrats/SNP/UK Government/UK Government Department of Health/HM Treasury Local Government Association
Government advisory body	Chief Medical Officer/Health Select Committee/Local Government Association
Health professional/Professional association	British Medical Association/Royal College of Physicians /Faculty of Public Health/NHS
Health charity/advocacy group University/academic researcher	University of Birmingham
Think-tank/commercial researcher	Adam Smith Institute/Institute of Economic Affairs/Institute for Fiscal Studies/TaxPayers’ Alliance
Retailer/retail association	Asda/Sainsbury’s/Tesco/British Retail Consortium/Scottish Retail Consortium/Scottish Grocers Federation
Manufacturer/associated industry or association	British Beer and Pub Association/JD Wetherspoon
International health organization EU Member State or EU body Police	

**Table 3:** Most polarizing concepts used and level of prominence in each debate.

*Rank as a polarizing concept in the MUP debate*		*Prominence^a^ MUP*	*Prominence SDIL*
Policy is supported by evidence	1	13	8
Responsibility deals with industry are ineffective	2	16	21
Policy will reduce consumption of commodity	3	1	3
*Policy will penalize responsible consumers^b^*	*4*	*6*	–
Policy is illegal	5	2	35
*Rank as a polarizing concept in the SDIL debate*		*Prominence SDIL*	*Prominence MUP*
Policy is supported by evidence	1	8	13
*Industry taking voluntary action^b^*	*2*	*5*	–
Policy will improve population health	3	4	3
Policy will reduce consumption of commodity	4	3	1
*Industry plays an active role in public health promotion^b^*	*5*	*13*	–

a Prominence indicates relative frequency of use in each debate (rank 1 = most frequently used);

b italics = concept unique to one network.

MUP = minimum unit pricing; SDIL = soft drinks industry levy.
