# Supplementary material for: Policy congruence and advocacy strategies in the discourse networks of minimum unit pricing for alcohol and the soft drinks industry levy
Source: Addiction. Author manuscript; Available in PMC 2021 Nov 15. (PMC7611988; doi:10.1111/add.15068)
Supplement: Data S1 [file EMS137637-supplement-Data_S1.docx]

| **Harmonisation of concept codes across the two debates – 63 in total, 29 are common to both**  **(blue indicates concepts common to both debates)** | |
| --- | --- |
| SDIL CONCEPT CODES | MUP CONCEPT CODES |
| *Advertising to young people is a particular issue* | *Advertising to young people is a particular issue* |
| Commodity consumption a particular problem for low-income groups | Cheap, easily available commodity drives specified health harm |
| Commodity consumption a particular problem for young people | *Commodity consumption causes health harm* |
| *Commodity consumption causes health harm* | *Commodity consumption costs public resources* |
| *Commodity consumption costs public resources* | *Commodity consumption falling, does not cause specified health harm* |
| *Commodity consumption falling, does not cause specified health harm* | Commodity consumption is/can be bad for society |
| *Commodity consumption problem too complex for fiscal solution* | *Commodity consumption problem too complex for fiscal solution* |
| *Commodity should be regulated like others that cause health harm* | Commodity misuse should be addressed by targetted measures |
| *Government action on commodity consumption is nanny statist* | *Commodity should be regulated like others that cause health harm* |
| *Government action required on commodity consumption* | *Government action on commodity consumption is nanny statist* |
| Government action required on commodity consumption in young people | *Government action required on commodity consumption* |
| *Greatest health harm from commodity consumption suffered by low-income groups* | *Greatest health harm from commodity consumption suffered by low-income groups* |
| Greatest health harm from commodity consumption suffered by young people | Heavist commodity consumers are price sensitive |
| Industry plays an active role in public health promotion | *Industry puts profit before public health* |
| *Industry puts profit before public health* | *Information and education needed to address commodity consumption* |
| *Information and education needed to address commodity consumption* | Majority consume responsibly |
| Other factors are important - parental control | *Other fiscal policies are preferable* |
| Other factors are important - reformulation | Policy could create a windfall for supermarkets |
| Other factors are important - retailer promotions | Policy could generate business for north of England |
| *Other fiscal policies are preferable* | Policy does not provide positive culture change in relation to commodity |
| *Policy is an inappropriate intervention in the market* | *Policy is an inappropriate intervention in the market* |
| Policy is badly designed | *Policy is illegal* |
| *Policy is illegal* | *Policy is regressive* |
| *Policy is regressive* | *Policy is supported by evidence* |
| *Policy is supported by evidence* | *Policy needed to address commodity "problem"* |
| Policy is too complex/costly to implement | *Policy needs to be part of a package of measures* |
| Policy is unfairly punitive | *Policy threshold needs to be set high enough to be effective* |
| *Policy needed to address commodity "problem"* | Policy will address youth consumption |
| *Policy needs to be part of a package of measures* | Policy will damage European industry interests |
| Policy sends a strong message | *Policy will damage industry and associated industries* |
| *Policy threshold needs to be set high enough to be effective* | *Policy will damage the wider economy* |
| Policy unnecessary - industry taking voluntary action (not due to PRD) | *Policy will have unintended adverse health consequences* |
| Policy will benefit the health of those on low income most | *Policy will improve population health* |
| *Policy will damage industry and associated industries* | Policy will increase illegal trade of commodity |
| *Policy will damage the wider economy* | Policy will penalise responsible consumers |
| *Policy will have unintended adverse health consequences* | Policy will protect some industry sectors |
| *Policy will improve population health* | *Policy will raise/save public resources* |
| *Policy will raise/save public resources* | Policy will reduce availability of cheapest commodity |
| *Policy will reduce consumption of commodity* | *Policy will reduce consumption of commodity* |
| *Population over-consumes commodity* | Policy will reduce crime |
| *Problematic consumption driven by social and economic conditions* | Policy will reduce social problems |
| Public supports government action | Population level measures are ineffective |
| *Public supports policy* | *Population over-consumes commodity* |
| *Responsibility deals with industry are ineffective* | *Problematic consumption driven by social and economic conditions* |
| There is a lifestyle related health crisis | *Public supports policy* |
| There is a lifestyle related health crisis for children | *Responsibility deals with industry are ineffective* |
