# Supplementary material for: Policy congruence and advocacy strategies in the discourse networks of minimum unit pricing for alcohol and the soft drinks industry levy
Source: Addiction. Author manuscript; Available in PMC 2021 Nov 15. (PMC7611988; doi:10.1111/add.15068)
Supplement: Data S2 [file EMS137637-supplement-Data_S2.doc]

| Stakeholder category | | SDIL Stakeholder organisations | MUP stakeholder organisations |
| --- | --- | --- | --- |
| Politician / Political Party |  | - Australian Government - Australian Greens - Brighton & Hove Council - Conservatives - Labour - Liberal Democrats - Liverpool City Council - Mexican politicians - National Action Party (PAN) - Plaid Cymru - Scottish Conservatives - Scottish Labour - Scottish Liberal Democrats - Scottish National Party - UK Government - UK Government: Dept for Environment, Food & Rural Affairs - UK Government: Dept of Health - UK Government: HM Treasury - UK Independence Party - Wolverhampton Council | - Conservatives - Labour - Liberal Democrats - Scottish Conservatives - Scottish Labour - Scottish Liberal Democrats - Scottish National Party - Scottish Government - UK Government - UK Government: Dept for Business - UK Government: Dept of Health - UK Government: HM Treasury - UK Government: Home Office |
| Government Advisory Body |  | - Behavioural Insights Team - Chief Medical Officer - Commons Health Select Committee - Food Responsibility Network - Food Standards Scotland - Local Government Association - Office for Budget Responsibility - Public Health England - Scientific Advisory Committee on Nutrition - Scotland's Chief Medical Officer - Scottish Food Commission - Scottish Health Survey - Scottish Parliament Information Centre | - Chief Medical Officer - Commons Health Select Committee - Local Government Association - Office for National Statistics - Office of Fair Trading |
| Health Professional / Professional Association |  | - Academy of Medical Royal Colleges - Association of Directors of Public Health - British Dental Association - British Dietetic Association - British Medical Association - East Lancashire NHS Trust - General Practitioners - NHS Confederation - NHS England - NHS Greater Glasgow and Clyde - Royal College of Dental Surgeons - Royal College of General Practitioners - Royal College of Midwives - Royal College of Paediatrics & Child Health - Royal College of Physicians - Royal College of Physicians Edinburgh - Royal College of Psychiatrists - Royal College of Surgeons Ireland - Royal Society for Public Health - UK Faculty of Public Health | - British Medical Association - Law Society - NHS Health Scotland - NHS England - Royal College of Nursing - Royal College of Physicians - UK Faculty of Public Health |
| Health Charity / Advocacy Group |  | - Alianza por la Salud Alimentaria   (Nutritional Health Alliance)   - British Heart Foundation - Cancer Research UK - Ceres - Child Growth Foundation - Children's Food Campaign - Diabetes UK - European Society of Lifestyle Medicine - Heart Research UK - Irish Heart Foundation - Jamie Oliver - National Obesity Forum - Nourish Scotland - Obesity Action Scotland - Obesity Health Alliance - Obesity Stakeholder Group - Soil Association - Sustain - The Richmond Group - UK Active - UK Health Forum - World Cancer Research Fund - Action on Sugar - World Obesity Federation | - Addaction - Alcohol Concern - Alcohol Focus Scotland - Alcohol Health Alliance - Alcohol Research UK - Balance - British Liver Trust - Drinkaware - Glasgow Council on Alcohol - Institute of Alcohol Studies - King's Fund - Scottish Health Action on Alcohol Problems - Thames Reach |
| University / Academic Researcher |  | - British Medical Journal - Brighton University - Centre for Dementia Prevention   (Edinburgh University)   - Centre for Diet and Activity Research (Cambridge University) - City University London - Cornell University - Duke University - Harvard School of Public Health - Karolinska Institute - King's College London - London Metropolitan University - National Institute of Public Health Mexico (INSP) - Queen Mary University - St Andrews University - Stirling University - Tufts University - University College London - University of Birmingham - University of California - University of Cambridge - University of Dundee - University of Glasgow - University of Liverpool - University of North Carolina - University of Oxford - University of Reading | - University of Birmingham - Centre for Addictions Research, University of Victoria - Curtin University - Glasgow Caledonian University - Newcastle University - University of Bedfordshire - University of Sheffield |
| Think tank/Commercial researcher |  | - Adam Smith Institute - Brighton Students Against Sugar Tax - Centre for Science in the Public Interest - Comres - DWF - El Poder del Consumidor - Ernst & Young - Euromonitor - Future Thinking - Institute for Fiscal Studies - Institute of Economic Affairs - Investec - Liberum - Marketing Sciences - McKinsey Global Institute - Numis - Oxford Economics - Shore Capital - Simon-Kucher & Partners - St Mary's Catholic school - Sucden Financial - TaxPayer's Alliance | - Adam Smith Institute - Campaign for Real Ale - Centre for Economic Business Research - Compecon - Institute for Fiscal Studies - Institute of Economic Affairs - NCB (stockbrokers) - TaxPayer’s Alliance |
| Retailer / Retail Association |  | - Aldi - Asda - British Retail Consortium - Lidl - Marks & Spencer - Sainsbury's - Scottish Grocers Federation - Scottish Retail Consortium - Tesco - Waitrose | - Asda - British Retail Consortium - Scottish Grocers Federation - Scottish Retail Consortium - Morrisons - Sainsbury’s - Tesco |
| Manufacturer / Associated Industry or Association |  | - AB Sugar - AG Barr - Abokado - Associated British Foods - Australian Beverages Council - British Beer & Pub Association - British Soft Drinks Association - British Sugar (part of AB Foods) - Britvic - Burger Brothers - Cadbury - Coca-Cola - Costa - Food and Drink Federation - Food and Drink Industry Ireland - Frankie & Benny's - Irish Beverage Council - Jamie Oliver Restaurant Group - JD Wetherspoon - Kellogg's - Leon - Lucozade Ribena Suntory - Mars Food - McDonalds - Mexican beverage Association - Moshimo - Nichols - Pepsi - Pizza Hut - Scotland Food and Drink - Sibberi - Starbucks - Sugar Nutrition UK - Tortilla - UK Sugar Bureau - Unilever - Union Jacks - Wild Orchid | - British Beer & Pub Association - Carlsberg UK - Chivas Brothers - Comite Vins - Diageo - European Spirits Organisation - Greene King - Heineken - JD Wetherspoon - Marston's - Mitchells & Butlers - Molson Coors - Portman Group - Punch Taverns - SAB Miller - Scotch Whisky Association - Scottish Beer and Pub Association - Scottish Licensed Trade Association - Tennent's - Wine and Spirit Trade Association |
| International Health Organisation |  | - National Institute of Pharmacy and Nutrition (Budapest) - Organisation for Economic Co-operation and Development - World Health Organisation |  |
| EU Member State or EU body |  |  | - Bulgaria - European Commission - France - Italy - Portugal - Spain |
| Police |  |  | - Police |
